# Trimethylamine-N-Oxide Pathway: A Potential Target for the Treatment of MAFLD

**DOI:** 10.3389/fmolb.2021.733507

**Published:** 2021-10-01

**Authors:** Xun Li, Jia Hong, Yao Wang, Maohua Pei, Luwen Wang, Zuojiong Gong

**Affiliations:** ^1^ Department of Infectious Diseases, Renmin Hospital of Wuhan University, Wuhan, China; ^2^ Department of Obstetrics and Gynaecology, Renmin Hospital of Wuhan University, Wuhan, China

**Keywords:** TMAO (trimethylamine oxide), MAFLD (metabolic associated fatty liver disease), trimethylamine, TMA precursor, FMO3, unfolded protein response, oxidative stress

## Abstract

Trimethylamine-N-oxide (TMAO) is a molecular metabolite derived from the gut flora, which has recently emerged as a candidate risk factor for metabolic dysfunction-associated fatty liver disease (MAFLD). TMAO is mainly derived from gut, where the gut microbiota converts TMA precursors into TMA, which is absorbed into the bloodstream through the intestinal mucosa, and then transformed into TMAO by hepatic flavin monooxygenases (FMOs) in the liver. High-nutrient diets rich in TMA precursors, such as red meat, eggs, and fish, are the main sources of TMAO. Excessively consuming such diets not only directly affects energy metabolism in liver, but also increases the concentration of TMAO in plasma, which promotes the development of MAFLD by affecting bile acid metabolism, unfolded protein response, and oxidative stress. In this review, we focused on the relationship between TMAO and MAFLD and summarized intervention strategies for reducing circulating TMAO concentration, aiming at providing new targets for the prevention and treatment of MAFLD.

## Introduction

Metabolic dysfunction-associated fatty liver disease (MAFLD), formerly named as non-alcoholic fatty liver disease (NAFLD), is characterized as excessive lipid accumulation within hepatocytes with overweight/obesity, presence of type 2 diabetes mellitus, or evidence of metabolic dysregulation (Eslam et al., 2020). With the increasing prevalence of obesity, diabetes mellitus and the metabolic syndrome, MAFLD has become the most frequent chronic liver disease worldwide (Vernon et al., 2011). It is estimated that the prevalence of MAFLD in the global general population is about 24% (Younossi et al., 2018; Huang et al., 2020). The mechanism of its pathogenesis is not fully understood, multiple parallel hits are believed to be involved, including insulin resistance, hormones secreted from the adipose tissue, nutritional factors, gut microbiota and genetic and epigenetic factors (Buzzetti et al., 2016).

In recent years, more and more attention has been paid to intestinal flora and its metabolites. There is a large microbial community in the human gut, and at least 20% of small molecules in the human blood are products of the microbiome, which plays a vital role in our physiology and metabolism (Rook et al., 2017). The intestinal microbiota regulates many metabolic processes in the host, including energy homeostasis, glucose metabolism, and lipid metabolism (Sonnenburg and Backhed, 2016). Trimethylamine-N-oxide (TMAO), a molecular metabolite derived from the gut flora, has recently emerged as a candidate risk factor for many chronic diseases including cardiovascular disease, kidney disease, type 2 diabetes mellitus, and MAFLD. In this review, we will focus on the role of TMAO in MAFLD and potential interventions strategies for reducing circulating TMAO concentration.

## The Source of TMAO

TMAO, a natural compound with the chemical formula of (CH3)3NO, is found naturally in large quantities in marine organisms (Zhang et al., 1999; Lidbury et al., 2014). It is a potent protein stabilizer that protects marine animals against the adverse effects of temperature, salinity, high urea, and hydrostatic pressure (Yancey et al., 2014; Velasquez et al., 2016). It can be degraded by endogenous enzymes of fish or bacterial enzymes into dimethylamine (DMA) or trimethylamine (TMA), its reduction has been suggested for estimating the quality of seafoods (Lundstrom and Racicot, 1983).

In human, TMAO is mainly derived from gut, where the gut microbiota converts dietary nutrients into TMA, which is absorbed into the bloodstream through the intestinal mucosa, and then transformed into TMAO by hepatic flavin monooxygenases (FMOs) in the liver (Zeisel and Warrier, 2017; Subramaniam and Fletcher, 2018). Diets rich in TMA precursors, such as choline and L-carnitine, are the main sources of TMAO (Zhang et al., 1999; Wang et al., 2011; Koeth et al., 2013). In addition, carnitine-related metabolites γ-butyrobetaine and trimethyllysine, the choline oxidation product betaine, as well as δ-valerobetaine in ruminant are also important TMA precursors (Koeth et al., 2014; Servillo et al., 2018; Li et al., 2019; Day-Walsh et al., 2021).

A study conducted by demonstrated that the rate of TMAO generation after single gavage with different precursors is L-carnitine > choline > betaine; However, long-term dietary intervention showed that the production for TMAO in chronic choline-treated group is 2.8 and 1.6 times than that in L-carnitine and betaine groups, respectively (Yu et al., 2020). Priscilla and colleagues also investigated TMA production from dietary precursors in an *in-vitro* model of the human colon, they found that the rate of TMA production from precursors is choline > L-carnitine > betaine > γ-butyrobetaine, and conversion of L-carnitine to TMA is slower than that of choline (Day-Walsh et al., 2021).

Meats (beef, veal, lamb, pork, ham, bacon), eggs, poultry and milk are rich in choline, while whole grains, fish, shellfish, beans and peas are other food sources of choline (Yonemori et al., 2013). Dietary carnitine is mainly found in foods of animal origin and lesser amounts are found in grains, fruits and vegetables (Steiber et al., 2004). A randomized-controlled dietary intervention study performed by Wang and colleagues found that chronic red meat, but not white meat or non-meat ingestion, increases plasma and urine TMAO; besides, compared to non-meat diets, red meat or white meat diets increase TMA and TMAO production from carnitine but not choline (Wang et al., 2019).

In addition to the TMA precursors mentioned above, TMAO itself is also an important source of TMAO in human. Fish is rich in TMAO, a randomized controlled trial in young men showed that fish yields higher circulating and urinary concentrations of TMAO, TMA, and DMA than eggs, beef, or fruit control (Cho et al., 2017). Siraphat et al. quantitatively elucidated the metabolic fate of orally consumed TMAO by using deuterium-labeled methyl d9-TMAO (d9-TMAO) in 40 healthy men, the results showed that plasma d9-TMAO could be detected as early as 15 min, increased until 1 h and remained elevated through the 6 h period after oral administration; the absorption of orally consumed TMAO is near complete and does not require processing by gut microbes (Taesuwan et al., 2017). Besides, TMAO is excreted mainly through the kidney, ∼96% of which is eliminated in urine by 24 h (Taesuwan et al., 2017).

## The Link Between TMAO and MAFLD

In recent years, due to the correlation between TMAO and cardiovascular disease (CVD) (Wang et al., 2011) and diabetes (Zhuang et al., 2019), the roles of TMAO in metabolic diseases have attracted extensive attention. The link between TMAO and MAFLD was first confirmed by a cross sectional observational study conducted by Chen and colleagues, they detected the plasm TMAO levels of healthy people and patients with MAFLD and found that the TMAO level of patients with MAFLD (0.434 μM) is 4.17 times higher than that of healthy controls (0.104 μM) (Chen et al., 2016). In a hospital-based case control study, Barrea and colleagues also found that high circulating TMAO levels are associated with obesity and the severity of MAFLD (Barrea et al., 2019). In fact, MAFLD is a metabolic liver disease that associated with obesity, insulin resistance (IR), diabetes, hypertension, hyperlipidaemia, and other metabolic disorders (Younossi, 2019). Except directly linked to MAFLD, other different metabolic disorders mediated by TMAO may also contribute to the development of MAFLD indirectly.

Obesity is a major risk factor for MAFLD, free fatty acids and metabolites released by adipocytes and cytokines secreted by macrophages in adipose tissue contribute to the of development MAFLD (Woo and Lavine, 2016). A meta-analysis showed a positive association between circulating TMAO and obesity. In addition, a dose-dependent relationship between circulating TMAO and obesity has been found in apparently healthy individuals (Dehghan et al., 2020). In a study of lab animals, Schugar et al. found that TMAO is associated with obesity and energy metabolism, antisense oligonucleotide-mediated knockdown or genetic deletion of the TMAO-producing enzyme FMO3 has a protective effect on the obesity of mice (Schugar et al., 2017).

There is a close relationship between IR/diabetes and MAFLD, the mechanisms by which IR promotes MAFLD include changes in rates of adipose tissue lipolysis and *de novo* lipogenesis, impaired mitochondrial fatty acid β-oxidation (FAO), changes in fat distribution, and alterations in levels of adipokines and cytokines (Tilg et al., 2017; Khan et al., 2019). A systemic review and dose-response meta-analysis found that plasma levels of TMAO in patients with diabetes are higher than in subjects without diabetes and there is a positive dose-dependent association between circulating TMAO levels and increased diabetes risk (Zhuang et al., 2019). In addition to humans, TMAO has also been shown to worsen impaired glucose tolerance in animals. Gao et al. investigated the effects of TMAO on glucose tolerance in high fat diet (HFD)-fed mice, they found that dietary TMAO exacerbates impaired glucose tolerance, blocks the insulin signaling pathway in the liver, and led to adipose tissue inflammation in mice (Gao et al., 2014).

Recently, a clinical study conducted by found that in obese patients, there is a positive correlation between TMAO levels and MAFLD histologic features; Besides, circulating TMAO levels are associated with NASH mainly in the presence of type 2 diabetes (Leon-Mimila et al., 2021). In addition to obesity, IR and diabetes, TMAO is also reported associated with other metabolic dysregulations that related to MAFLD, including dyslipidemia (Barrea et al., 2018) and hypertension (Ge et al., 2020).

## The Underlying Mechanisms of TMAO in the Development of MAFLD

**FIGURE 1:**
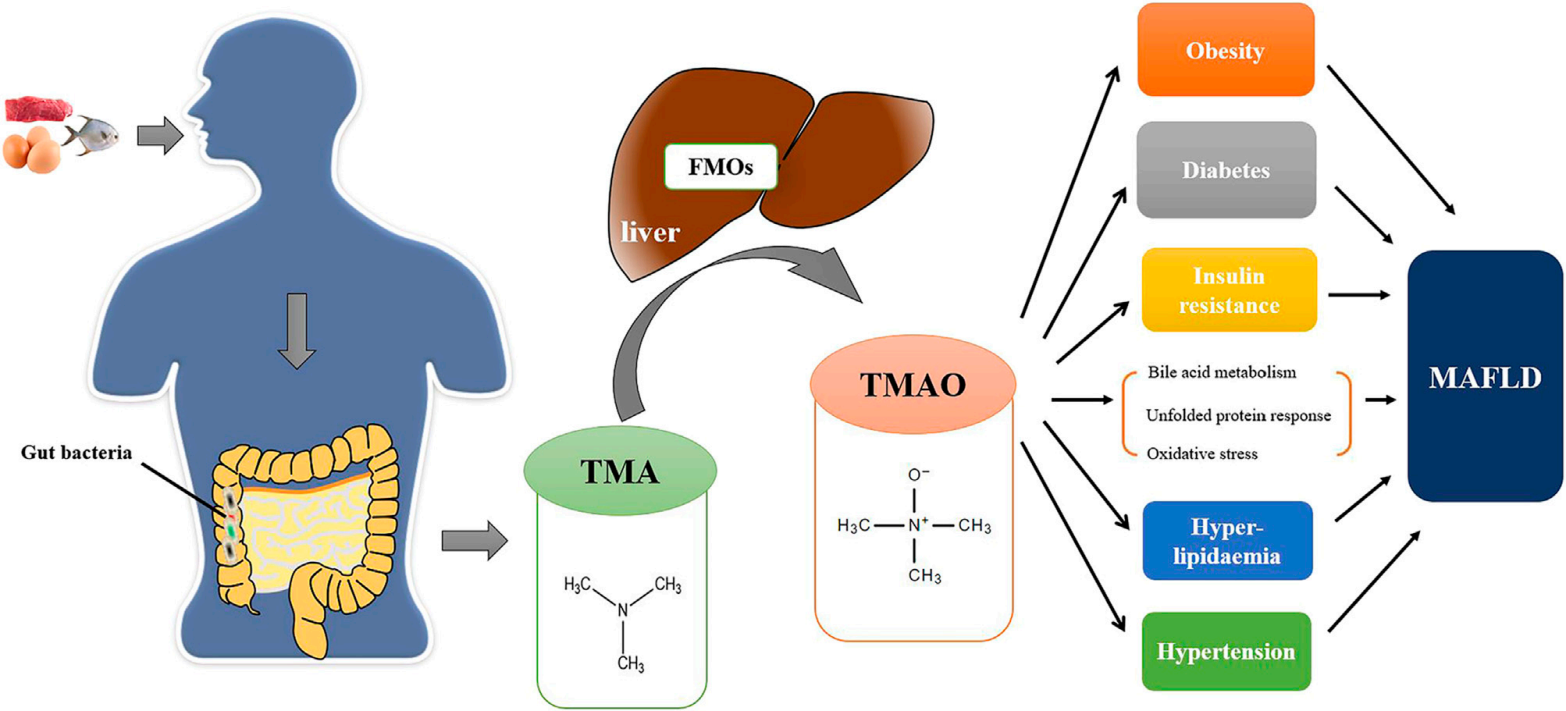
The TMAO pathway in the pathogenesis of MAFLD. Foods (e.g., red meat, eggs, fish, etc.) rich in TMA precursors are digested as choline, L-carnitine, betaine and γ-butyrobetaine in the digestive tract. Excessive TMA precursors that cannot be absorbed are metabolized to TMA by gut bacteria in colon. TMA is then absorbed into the bloodstream through the intestinal mucosa and transformed into TMAO by FMO1 and FMO3 in the liver. TMAO can directly promote the development of MAFLD by affecting bile acid metabolism, unfolded protein reaction, and oxidative stress and indirectly affect the progression of MAFLD by other metabolic disorders (e.g., obesity, diabetes, insulin resistance, etc.).

Although TMAO has been shown to be linked to MAFLD, the molecular mechanisms of TMAO remain obscure. TMAO may contribute to the development of MAFLD through the following mechanisms (Figure 1).

### Bile Acid Metabolism

Bile acids (BAs) are amphiphilic steroid molecules synthesized from cholesterol in the liver. BAs function as signaling molecules through various receptors, including farnesoid X receptor (FXR), vitamin D receptor, pregnane X receptor and members of the G-protein-coupled receptor superfamily, to regulate their own synthesis as well as other metabolic processes, such as glucose, lipid and energy metabolism (Arab et al., 2017). Dysregulated BA metabolism is an important indicator in the pathology of MAFLD, which directly affects lipid metabolism, immune environment, and intestinal bacteria (Yu et al., 2018).

An animal study conducted by Koeth and colleagues found that TMAO decreases the bile acid pool size and inhibits the expressions of the key BA synthetic enzymes (Cyp7a1 and Cyp27a1) and BA transporters (Oatp1, Oatp4, Mrp2, and Ntcp) in the liver (Koeth et al., 2013). Another animal study showed that inhibition of TMAO formation can upregulate the host hepatic bile acid synthetic enzyme CYP7A1 and alter the expression of hepatic genes critical for bile acid feedback regulation, thus preventing diet-driven hepatic cholesterol accumulation (Pathak et al., 2020).

Tan et al. (2019) demonstrated that serum levels of TMAO are positively correlated with the serum levels of total BA in patients with MAFLD by conducting a case-control study, and administration of TMAO impairs liver function and increases hepatic triglyceride accumulation and lipogenesis in a murine model; The mechanism is related to the regulation of bile acid metabolism and the inhibition of FXR signaling pathway by TMAO.

### Unfolded Protein Response

Another underlying mechanism of TMAO in the development of MAFLD maybe unfolded protein response (UPR). UPR is a highly conserved pathway that monitors endoplasmic reticulum (ER) proteostasis, allowing cells to manage ER stress (Hetz et al., 2020). It is an adaptive signaling pathway that reduces protein synthesis and activates specific protein folding and degradation pathways to restore endoplasmic reticulum homeostasis by controlling transcriptional activation of a range of target genes (Moncan et al., 2021). When endoplasmic reticulum homeostasis can’t be maintained, sustained UPR induces apoptosis or changes in cell function (Ren et al., 2021). Sustained UPR can promote the development of MAFLD by regulating lipogenesis, causing inflammation, and inducing hepatocyte death (Lebeaupin et al., 2018; Song and Malhi, 2019).

Research conducted by Chen et al. indicated that endoplasmic reticulum stress kinase PERK (EIF2AK3) is a receptor for TMAO; After binding with PERK, TMAO selectively activates PERK-mediated UPR, thereby promoting metabolic dysfunction (Chen et al., 2019). In addition, Govindarajulu and colleagues also demonstrated that TMAO induces deficits in synaptic plasticity through the ER stress-mediated PERK signaling pathway in a mice model (Govindarajulu et al., 2020).

Of note, TMAO in the pathogenesis of Alzheimer’s disease (AD) indicated that PERK is not the only mechanism by which TMAO induces UPR. AD is characterized by the accumulation and aggregation of misfolded proteins in brain, the sustained activation of the UPR mediated by the misfold proteins initiates or mediates neurodegeneration (Hoozemans et al., 2005). In a hospital-based case control study, Vogt et al. (2018) found that elevated cerebrospinal fluid (CSF) TMAO is associated with biomarkers of AD pathology (phosphorylated tau and phosphorylated tau/Aβ) and neuronal degeneration (total tau and neurofilament light chain protein). Down-regulation of TMAO levels by dimethyl-1-butanol (DMB), a TMA formation inhibitor, is able to ameliorate the cognitive deterioration and long-term potentiation (LTP) in APP/PS1 mice and decrease amyloid-β (Aβ)1-42, β-secretase, and β-secretase-cleaved C-terminal fragment (βCTF) levels in the hippocampus (Gao et al., 2019). These studies implied TMAO-mediated aggregation of unfolded or misfolded proteins may be another mechanism linking TMAO to UPR. However, it’s perplexing that TMAO used to act as a chemical molecular chaperone that helps the proper targeting and function of misfolded proteins (Morello et al., 2000).

### Oxidative Stress

Oxidative stress is a condition in which the production of reactive oxygen species (ROS) exceeds the detoxifying power of antioxidants, it contributes significantly to the pathogenesis and progression of MAFLD (Arroyave-Ospina et al., 2021). It is reported that ROS drives MAFLD progression through reprogramming of hepatic lipid metabolism, changing insulin sensitivity, and modulating inflammatory responses (Chen et al., 2020). Currently, there is a lack of evidence for the involvement of oxidative stress in the pathogenesis of MAFLD mediated by TMAO, but studies on cardiovascular diseases suggest that TMAO is associated with oxidative stress. Brunt and colleagues explored the effect and underlying mechanism of TMAO on endothelial dysfunction, they found that TMAO directly impairs vascular endothelial function via superoxide-driven oxidative stress, which is not associated with increased expression of NADPHO, mitochondrial superoxide production or superoxide dismutase (SOD) -related endogenous antioxidant defenses (Brunt et al., 2020). Similar results were also confirmed by Ke and colleagues in another study (Ke et al., 2018).

## Intervention Strategies to Reduce TMAO Concentrations

### Changing Dietary Habits

**FIGURE 2:**
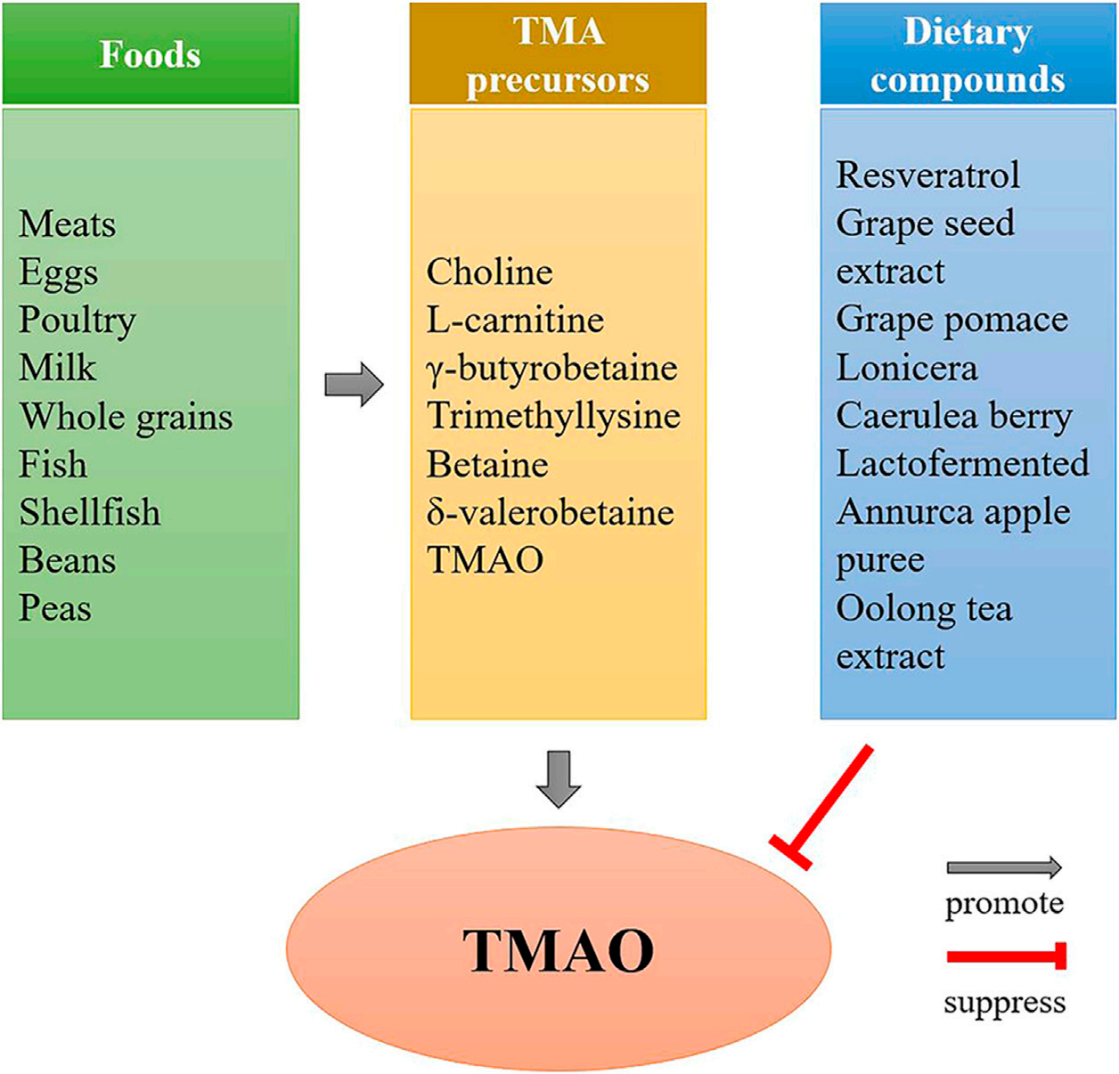
The effect of diet on TMAO formation. Diets rich in TMA precursors, such as red meat, eggs, fish, etc., are the main source of TMAO, while the TMAO concentrations can be reduced by some dietary compounds, such as resveratrol, grape seed extract, etc.

TMAO is mainly derived from the gut, and its concentration depends on the type and amount of dietary nutrients consumed (Figure 2). Although diets rich in choline, L-carnitine, γ-butyrobetaine, trimethyllysine and betaine are the main sources of TMAO, total intake is also critical for TMAO concentration. Eggs are major sources of dietary choline, researchers explored the relationship between plasma TMAO concentrations and consumption of eggs, they found that participants consumed two eggs per day increases plasma choline without increasing TMAO concentrations (Missimer et al., 2018), while consumption of ≥2 egg yolks result in an increased formation of TMAO (Miller et al., 2014). Choline is an essential nutrient which is absorbed in the upper small intestine by specific enterocyte transporters via facilitated (Radziejewska and Chmurzynska, 2019), only excessive amounts are metabolized into TMA by the gut microbiota in the colon (Papandreou et al., 2020).

In addition to be influenced by total intake of TMA precursors, TMAO levels can also be reduced by some bioactive compounds in nutrients. Studies have suggested that some dietary compounds, such as resveratrol (Chen et al., 2016), grape seed extract (Zhao et al., 2019), grape pomace (Annunziata et al., 2019), lonicera caerulea berry (Liu et al., 2018), lactofermented Annurca apple puree (Tenore et al., 2019) and Oolong tea extract (P. Y. Chen et al., 2019), are able to reduce TMAO concentrations in human or animals.

### Regulating the Intestinal Flora

Intestinal flora is a key determinant of TMA production. TMAO concentrations are associated with the overall microbial compositions (Li et al., 2021). By conducting a cross-sectional analysis of data collected from 1,653 participants, Fu et al. found that plasma TMAO is positively associated with abundance of 12 bacterial genera in fecal, including three *Bacteroidetes* (Prevotella 7, Prevotella 2, an uncultured Prevotellaceae); five Firmicutes [Mitsuokella, *Ruminococcaceae* NK4A214 group (*Ruminococcus*) torques group, (*Bacteroides*) pectinophilus group, *Eisenbergiella*]; three Proteobacteria (*Bilophila*, *Desulfovibrio*, uncultured *Rhodospirillales*); and one Fusobacteria (*Fusobacterium*) (Fu et al., 2020). Altering the composition of the gut microbiota by taking probiotics or antibiotics has been shown to be a candidate method for reducing TMAO concentrations.

Many different types of probiotics have been used to try to reduce TMAO concentrations, but only a few have been shown to be effective. Qiu et al. administered five potentially probiotic strains supplemented with 1.3% choline to mice, they found that *Lactobacillus plantarum* ZDY04 can decrease serum TMAO and cecal TMA levels by modulating the relative abundance of the families *Lachnospiraceae*, *Erysipelotrichaceae* and *Bacteroidaceae* and the genus *Mucispirillum* (Qiu et al., 2018). Besides, *Enterobacter aerogenes* ZDY01 and *Lactobacillus casei* have also been shown to reduce circulating TMAO levels in murine model (Qiu et al., 2017; Hsu et al., 2019).

In addition to probiotics, antibiotics are also candidates for lowering TMAO levels. It is reported that after administrated with metronidazole (500 mg twice daily) plus ciprofloxacin (500 mg once daily) for 1 week, near-complete suppression of detectable TMAO and d9-TMAO are observed under the challenge of phosphatidylcholine in both plasma and urine (Tang et al., 2013). Another study proved that daily administration of vancomycin 100 mg/ kg, neomycin 200 mg/ kg, metronidazole 200 mg/ kg plus ampicillin 200 mg/ kg are able to decrease plasm TMAO levels in rats (Zhang et al., 2020). Other antibiotics that may reduce TMAO levels including amoxicillin/clavulanate, clarithromycin and ceftriaxone (Ottiger et al., 2016).

### Application of TMA Formation Inhibitors

**FIGURE 3:**
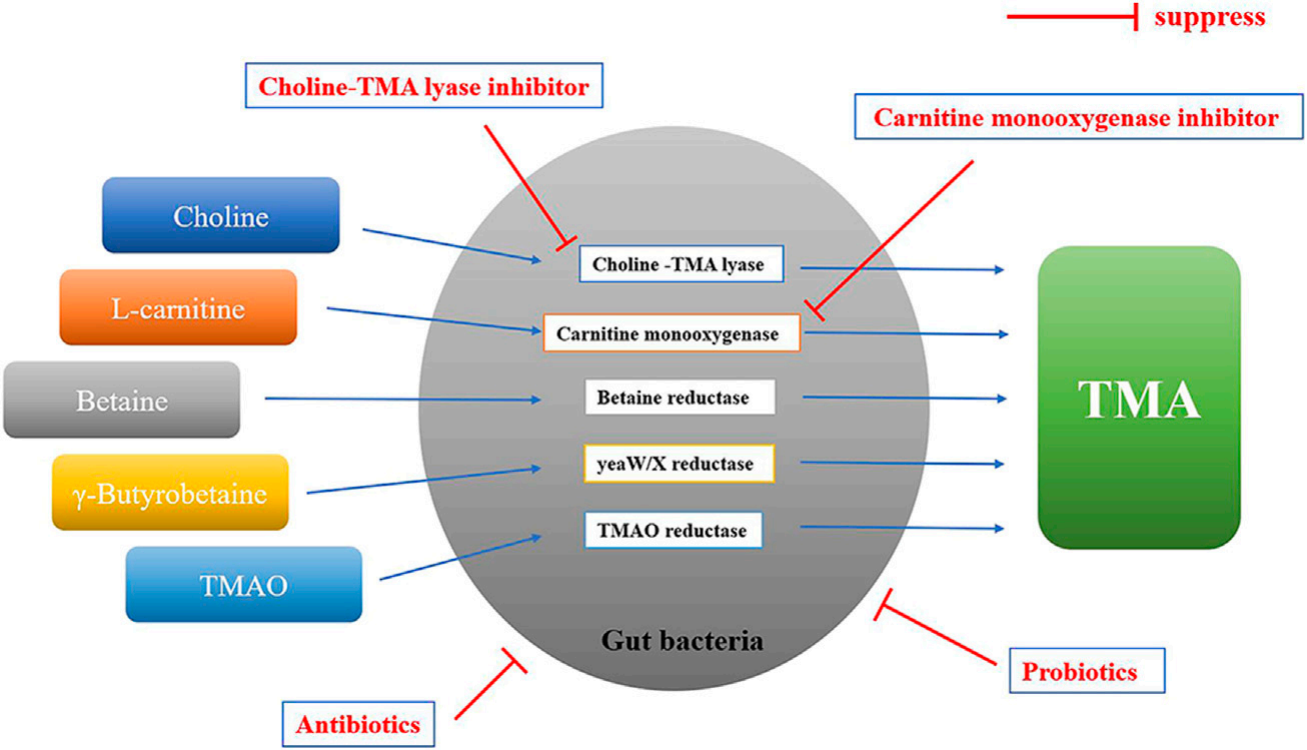
The formation of TMA in gut bacteria. TMA precursors are converted to TMA by different enzyme systems in gut bacteria, including choline-TMA lyase (cutC/D), carnitine monooxygenase (cntA/B), yeaW/X TMA lyase, betaine reductase and TMAO reductase. Gut bacteria involved in the formation of TMA: *Bacteroidetes*, Firmicutes, Proteobacteria, Fusobacteria, etc.; Probiotics: Lactobacillus plantarum ZDY04, aerogenes ZDY01, Lactobacillus casei, etc.; Antibiotics: metronidazole plus ciprofloxacin, or vancomycin, neomycin, metronidazole plus ampicillin, etc.; Choline-TMA lyase inhibitor: DMB, IMC, FMC, etc.; Carnitine monooxygenase inhibitor: MMV3, etc.

In bacteria, there are several different enzyme systems involved in the conversion of TMA precursors to TMA that have been identified, including choline-TMA lyase (cutC/D), carnitine monooxygenase (cntA/B), yeaW/X TMA lyase, betaine reductase and TMAO reductase (Figure 3). The choline utilization (cut) gene cluster was first identified by Balskus and colleagues, which transforms choline into TMA via gene products of cutC and cutD (Craciun and Balskus, 2012). The carnitine monooxygenase (CntA) and associated reductase (CntB) are responsible for carnitine metabolism to TMA in representative genomes of the human microbiota (Zhu et al., 2014). yeaW/X TMA lyase is a microbial enzyme complex that produces TMA directly from γ-butyrobetaine and other TMA precursors including L-carnitine, choline, and betaine (Koeth et al., 2014). While betaine and TMAO reductase are responsible for converting betaine and TMAO to TMA, respectively (Zuo et al., 2020). Among these enzyme systems, choline-TMA lyase and carnitine monooxygenase are the key enzymes (Rath et al., 2017), which are the targets for reducing TMAO concentrations.

The first choline-TMA lyase inhibitor, DMB, was identified by Wang and colleagues, which is shown to non-lethally inhibit distinct microbial TMA lyases, and reduce TMAO levels in mice fed a high-choline or L-carnitine diet (Wang et al., 2015). Subsequent studies also confirmed that DMB can improve certain diseases by reducing TMAO concentrations, such as allogenic gain-versus-host disease (Wu et al., 2020) and endothelial dysfunction (Brunt et al., 2020). In addition to DMB, two other choline-TMA lyase inhibitors, iodomethylcholine (IMC) and fluoromethylcholine (FMC), are also proved be able to induce an almost complete reduction in plasma TMA and TMAO levels following a single oral dose for a sustained period (Roberts et al., 2018).

Compared to cutC/D, the identification of CntA/B inhibitor is relatively lagging. More recently, Mussa et al. reported the first structures of the carnitine oxygenase CntA and identified MMV3 as a potential inhibitor of CntA; The formation of TMA has almost been completely inhibited by MMV3 in *A. baumannii* cells pretreated with carnitine (Quareshy et al., 2020).

### Application of FMOs Inhibitors

FMOs are monooxygenase that oxygenates nucleophilic heteroatom-containing chemicals and drugs and converts them into harmless, polar metabolites that are easily excreted (Cashman and Zhang, 2006). They utilize the reducing equivalents of NADPH to reduce one atom of molecular oxygen to water, while the other atom is used to oxidize the substrate (Krueger and Williams, 2005). There are five subtypes of FMOs in human, including FMO1 ∼FMO5. The elucidation of the pathogenesis of trimethylaminuria, a fish malodor syndrome, indicates that FMO3 is involved in TMA metabolism (Treacy et al., 1998). In fact, FMO1 is also participated in the oxidization of TMA to TMAO, however, its specific activity is only 1/10 that of FMO3 (Bennett et al., 2013).

Numerous studies indicate that inhibition of FMOs, especially FMO3, is an effective target for reducing TMAO concentrations (Bennett et al., 2013; Zhu et al., 2018; Chen et al., 2019; Shih et al., 2019; Chen et al., 2019). But limited by the availability of specific inhibitors, most of these studies are performed using genetic interference techniques. Some chemical components that metabolized by FMOs are currently regarded as potential inhibitors of TMA oxidation. Methimazole, an anti-thyroid drug, is a high-affinity substrate of FMO3, which competitively suppress the metabolism of other compounds (Gao et al., 2018). A study conducted by Heidi et al. showed that TMAO levels increases in mice fed with L-carnitine, the effect is inhibited by methimazole treatment with a dose at 15 mg/ kg (Collins et al., 2016). Indole-3-carbinol and its acid condensation product 3,3′-Diindolylmethane, which are also metabolized by FMOs, have been shown to suppress the activities of FMOs and reduce TMAO concentrations (Cashman et al., 1999; Chen et al., 2019).

### Other Intervention Strategies

In addition to the intervention strategies mentioned above, other strategies can also be used as potential approaches. Some traditional Chinese medicine are proved be able to lower TMAO concentrations, such as Alisma orientalis beverage (Zhu et al., 2020), Ginkgolide B (Lv et al., 2021), Berberine (Li et al., 2021), etc. Besides, vitamins are other candidates for reducing TMAO concentrations, such as vitamin B and vitamin D (Obeid et al., 2017).

## Conclusions and Perspective

MAFLD is a metabolic disease that closely related with dietary patterns. Excessively consuming high-nutrient diets, such as red meat, eggs, and fish, not only directly affects energy metabolism, but also regulates the composition of intestinal flora, leading to an increase in plasma TMAO concentration. In last years, the relationship between TMAO and metabolic diseases has attracted more and more attention. TMAO has emerged as an attractive serum marker and therapeutic target of metabolic diseases. Although there are some links between TMAO and MAFLD and administration of TMAO impairs liver function and increases hepatic triglyceride accumulation and lipogenesis in animal models, whether reducing TMAO concentrations could prevent MAFLD remains unclear due to lack of relevant studies. In this review, we proposed that TMAO may promote the pathogenesis of MAFLD through bile acid metabolism, unfolded protein reaction, and oxidative stress. However, there is still a lack of direct evidence for these mechanisms except bile acid metabolism. More importantly, as a natural chemical molecule, the targets of TMAO *in vivo* are not fully elucidated. Especially in UPR, although TMAO can selectively activate PERK-mediated UPR by binding to PERK, this does not explain TMAO-mediated aggregation of unfold or misfolded proteins in AD. Therefore, more studies are needed to confirm the role and specific mechanisms of TMAO in MAFLD. Notably, an analysis of hepatic gene expression of MAFLD patients showed that the expression of FMO1 but not FMO3 increases in MAFLD (Arendt et al., 2015), whether this is related to TMAO needs to be further confirmed.

## Abbreviations

Aβ, amyloid-β; BA, bile acid; CVD, cardiovascular disease; CSF, cerebrospinal fluid; DMA, dimethylamine; DMB, dimethyl-1-butanol; FAO, fatty acid β‐oxidation; FMC, fluoromethylcholine; FMO, hepatic flavin monooxygenase; FXR, farnesoid X receptor; HFD, high fat diet; IMC, iodomethylcholine; LTP, long-term potentiation; MAFLD, metabolic dysfunction-associated fatty liver disease; NAFLD, non-alcoholic fatty liver disease; ROS, reactive oxygen species; SOD, superoxide dismutase; TMAO, trimethylamine-N-oxide; TMA, trimethylamine; UPR, unfolded protein response; βCTF, β-secretase-cleaved C-terminal fragment.
